# Reading Self-Perceived Ability, Enjoyment and Achievement: A Genetically Informative Study of Their Reciprocal Links Over Time

**DOI:** 10.1037/dev0000209

**Published:** 2017-04

**Authors:** Margherita Malanchini, Zhe Wang, Ivan Voronin, Victoria J. Schenker, Robert Plomin, Stephen A. Petrill, Yulia Kovas

**Affiliations:** 1 Psychology Department, Goldsmiths University of London, and International Centre for Research in Human Development, Tomsk State University; 2 Center for Gerontology, Virginia Tech; 3 Psychological Institute of Russian Academy of Education, and Laboratory for Cognitive Investigations and Behavioural Genetics, Tomsk State University; 4 Department of Psychology, The Ohio State University; 5 MRC Social, Genetic, & Developmental Psychiatry Centre, Institute of Psychiatry, King’s College London; 6 Department of Psychology, The Ohio State University; 7 Psychology Department, Goldsmiths University of London, and Laboratory for Cognitive Investigations and Behavioural Genetics, Tomsk State University

**Keywords:** reading, reading motivation, longitudinal, behavioral genetics

## Abstract

Extant literature has established a consistent association between aspects of reading motivation, such as enjoyment and self-perceived ability, and reading achievement, in that more motivated readers are generally more skilled readers. However, the developmental etiology of this relation is yet to be investigated. The present study explores the development of the motivation–achievement association and its genetic and environmental underpinnings. Applying cross-lagged design in a sample of 13,825 twins, we examined the relative contribution of genetic and environmental factors to the association between reading enjoyment and self-perceived ability and reading achievement. Children completed a reading comprehension task and self-reported their reading enjoyment and perceived ability twice in middle childhood: when they were 9–10 and 12 years old. Results showed a modest reciprocal association over time between reading motivation (enjoyment and perceived ability) and reading achievement. Reading motivation at age 9–10 statistically predicted the development of later achievement, and similarly, reading achievement at age 9–10 predicted the development of later motivation. This reciprocal association was observed beyond the stability of the variables and their contemporaneous correlation and was largely explained by genetic factors.

Good reading ability is crucial in modern literate society. It has a fundamental role in how we acquire knowledge and has been associated with employment level and socioeconomic status (e.g., Ritchie & Bates, 2013). Reading is also a cultural activity that many enjoy. There are vast individual differences in reading ability, partly attributable to cognitive skills such as verbal IQ and phoneme awareness (e.g., Oakhill & Cain, 2012; Warmington & Hulme, 2012). In addition, research suggests that reading motivation is related to the development of reading, above and beyond the effects of cognitive abilities (Morgan & Fuchs, 2007; Wigfield & Eccles, 2000).

Reading motivation refers to beliefs, attitudes, and values individuals hold specific to reading activities (Ryan & Deci, 2000; Wigfield, 1997). Two aspects of motivation that have received much attention are reading enjoyment and reading self-perceived ability. Reading enjoyment indicates pleasure gained from a reading activity (Wigfield, 1997). Children may enjoy reading for many different reasons, including curiosity and eagerness for intellectual development and positive feedback on their reading skills. Enjoyment of reading is associated with frequent reading activities, intense concentration during reading, and better reading performance (Baker & Wigfield, 1999; De Naeghel, Van Keer, Vansteenkiste, & Rosseel, 2012; Wigfield & Guthrie, 1997).

Reading self-perceived abilities—individuals’ perceptions of their reading competence (Wigfield, 1997)—are also positively associated with objectively measured reading performance (Baker & Wigfield, 1999; Greven, Harlaar, Kovas, Chamorro-Premuzic, & Plomin, 2009; Guay, Marsh, & Boivin, 2003; Morgan & Fuchs, 2007). In addition, reading self-perceived ability is positively related to how much children read in and out of school, how much they enjoy reading, how likely they are to choose more challenging reading materials, and their effort and perseverance when facing difficult reading tasks (Baker & Wigfield, 1999; Linnenbrink & Pintrich, 2003; Stipek, 1996).

## Longitudinal Associations Between Reading Achievement and Reading Motivation

Although several studies report modest to moderate correlations between reading achievement and several aspects of reading motivation, the findings are mixed with respect to the developmental nature of this association (e.g., Baker & Wigfield, 1999; Guthrie et al., 2006). One unresolved issue is how the motivation–achievement association develops. Several theories have been put forward addressing the causal ordering in the emergence of the motivation–achievement relationship. Early theories of the association between achievement and motivation favored unidirectional approaches. Two contrasting early theoretical frameworks are the Self-Enhancement Model and the Skill Development Model.

According to the Self-Enhancement Model, individual differences in motivation influence subsequent development of academic performance (Calsyn & Kenny, 1977). Confident and interested readers are more invested in learning and mastering reading skills through frequent reading, and this frequent print exposure further results in better reading skills (Calsyn & Kenny, 1977). Support for this model comes from early educational experimental programs, demonstrating that interventions designed to increase motivation lead to significant improvements in children’s reading ability (e.g., Guthrie et al., 1996, 2006; Wigfield, Guthrie, Tonks, & Perencevich, 2004). However, most of these studies did not consider the potential link from achievement to motivation.

This influence of achievement on subsequent motivation is central to the Skill Development Model (Calsyn & Kenny, 1977). For example, children at risk of reading failure are more likely to encounter difficulty and frustration in their early reading experiences, which may in turn lead to decreased motivation to read. The support for this model has been inconsistent. For example, one intervention study failed to observe improvements in children’s reading motivation as a consequence of improved reading skills in a group of unskilled readers (Morgan, Fuchs, Compton, Cordray, & Fuchs, 2008). However, several longitudinal studies have supported the temporal precedence of achievement in the reading motivation–achievement relationship in samples of several ages—from early elementary school to middle school ages (e.g., Aunola, Leskinen, Onatsu-Arvilommi, & Nurmi, 2002; Chapman & Tunmer, 1997; Skaalvik & Valas, 1999). These studies utilized cross-lagged longitudinal analyses in which the longitudinal effect of one construct on another is estimated beyond the stability of each construct and the concurrent correlation between constructs. Specifically, these studies demonstrated that individual differences in children’s reading performance predicted subsequent variation in children’s reading motivation, whereas reading motivation failed to predict subsequent reading performance (Aunola et al., 2002; Chapman & Tunmer, 1997; Skaalvik & Valas, 1999). However, these studies involved relatively small samples and may have been underpowered to detect reciprocal links between reading motivation and achievement.

The reciprocal relationship is central to a third theoretical framework, according to which achievement and motivation have a mutual influence on one another (Morgan & Fuchs, 2007). The reciprocal model has been supported by longitudinal studies that have explored the motivation–achievement relation in several academic domains including literacy and mathematics (e.g., Guay et al., 2003; Luo, Haworth, & Plomin, 2010; Marsh & Martin, 2011; Muijs, 1997).

Several methodological differences may explain the inconsistencies found among previous studies with respect to the temporal and causal ordering between reading achievement and reading motivation. Differences in sample size and sample characteristics, study design, and statistical methods could all contribute to the discrepancies in the literature. For example, some studies examined children in the normal range of reading ability (e.g., Guthrie et al., 1996), whereas others focused on poor readers (e.g., Morgan et al., 2008). Some studies used experimental designs but only examined immediate or short-term outcomes (e.g., Guthrie et al., 1996), while others relied on correlational designs to investigate longer-term outcomes (e.g., Marsh & Martin, 2011).

## Genetic and Environmental Etiology

Examining the genetic and environmental etiology of the longitudinal links between reading motivation and reading achievement can provide new insights into processes through which the two constructs interact. Research exploring factors contributing to variation in academic motivation and its association with achievement has largely focused on the role of environments (Deci & Ryan, 2008; Stipek, 1996; Wigfield & Eccles, 2000). In particular, family environment, relationships with parents, parents’ and teachers’ educational expectations and attitudes, teachers’ instructional style and quality, and teacher–student and peer relationships have all been found to be important for academic motivation (Deci & Ryan, 2008; Stipek, 1996; Wigfield & Eccles, 2000). A number of recent studies, using genetically informative approaches, have demonstrated that genetic factors are also involved in explaining individual differences in academic motivation (Kovas et al., 2015).

For example, a recent international twin study of over 13,000 children demonstrated that genetic factors account for approximately 40% of individual differences in self-perceived ability and enjoyment of learning in numerous academic domains, including language, mathematics, and science (Kovas et al., 2015). This was consistent across a wide age range and across six countries that were included in the study. Environmental influences stemmed entirely from unique individual experiences and did not contribute to similarity in academic motivation in children raised in the same family. This study suggests that resemblance among family members in academic motivation is entirely attributable to genetic influences, whereas dissimilarities among family members are largely explained by individual specific environmental factors. Even objectively shared environments, such as family educational resources and classroom environments, seem to be nonshared in terms of the actual experience.

Several studies examined the genetic and environmental etiology of the concurrent and longitudinal relations between academic motivation and academic achievement. For example, in a sample of 13-year-old twins from Germany, the contemporaneous correlations between motivation and academic performance in language and mathematics were mostly explained by genetic factors (Gottschling, Spengler, Spinath, & Spinath, 2012). In the large U.K. Twins Early Development Study (TEDS), academic self-perceived ability and overall academic performance of 9-year old children correlated primarily for genetic reasons (Greven et al., 2009). The study also found that the link from self-perceived ability at age 9 to achievement at age 12 was mostly explained by genetic factors.

Using the same TEDS sample, Luo et al. (2010) examined the longitudinal cross-lagged relations between a domain general composite of self-perceived ability and academic performance between ages of 9 and 12. In line with the reciprocal model, modest mutual links were found between domain general academic motivation and achievement. These cross-lagged reciprocal relations were mediated largely through genetic pathways (Luo et al., 2010). Only one study has examined the etiology of the reciprocal association between motivation and achievement in a domain specific context. This investigation, also using TEDS data, explored the cross-lagged associations between motivation and achievement specific to mathematics (Luo, Kovas, Haworth, & Plomin, 2011). The prediction from teacher-rated mathematics achievement at age 9 to subsequent mathematics motivation at age 12 was attributable to genetic factors, whereas the link from early motivation to subsequent achievement was mediated through both genetic and child-specific environmental pathways (Luo et al., 2011).

Overall, findings from genetically informative twin studies point to the importance of genetic influences and child-specific environmental experiences in the etiology of academic motivation in diverse academic domains. Shared environmental factors are found to have negligible effects on individual differences in academic motivation. Additionally, although the longitudinal association between domain general motivation and achievement is largely mediated by genetic factors, the domain-specific association between mathematics achievement and motivation is affected by both genetic and nonshared environmental factors. These differences in the etiology of longitudinal links in domain-general versus mathematics specific achievement and motivation suggest potential differences in the underlying mechanisms and provide rationale for the study of other specific domains, such as reading. The present study used a genetically sensitive cross-lagged approach to explore the longitudinal association between reading motivation and reading achievement. Based on the existing literature summarized above, we propose the following hypotheses:

1 Reciprocal longitudinal links of similar strength exist between reading motivation (enjoyment and self-perceived ability) and reading achievement.
2 Similar to the domain of mathematics, both genetic and nonshared environmental factors contribute to the observed longitudinal cross-lagged associations between reading motivation and reading achievement.

## Method

### Participants

Participants (*N* = 13,825) are members of the Twins Early Development Study (TEDS), a population-based longitudinal study of twins that focuses on the longitudinal relations of cognitive and behavioral traits from infancy to young adulthood. Over 15,000 families from England and Wales with twins born between 1994 and 1996 have participated over the years (Haworth, Davis, & Plomin, 2013). The families in TEDS are representative of the British population in their socioeconomic distribution, ethnicity, and parental occupation (Oliver & Plomin, 2007).

The present study included two waves of data collection. The first wave took place when the twins were between the ages of 9 and 10, and the second wave when the twins were 12 years old. For each wave of data collection, children completed a series of questionnaires and cognitive assessments online. In total, data from 6927 twin pairs (2502 MZ pairs and 4425 DZ pairs; 53% female) were used in the current investigation, excluding those who had reported medical or neurological conditions. Sample sizes varied across time and measures, and details regarding the sample size for each measure can be found in Table 1. Data collections at age 9/10 and age 12 received approval by the Institute of Psychiatry ethics committee.[Table-anchor tbl1]

### Measures

#### Reading motivation: reading enjoyment and self-perceived ability

At age 9/10 and age 12, the twins completed a series of questionnaires about their attitudes toward several academic subjects. Two items assessed their motivation for reading (National Assessment of Educational Progress, 2003). The first item measured reading self-perceived ability: “How good do you think you are at reading?” rated on a scale from 1 to 5, with 1 = *very good* and 5 = *not at all good*. The second item measured reading enjoyment: “How much do you like reading?” rated on a scale from 1 to 5, with 1 = *very much* and 5 = *not at all*. The scores for reading self-perceived ability and reading enjoyment were moderately correlated at both waves (*r* = .54 and .57 at age 9/10 and age 12, respectively). As the two aspects of reading motivation are conceptually distinct, we conducted the analyses on the reading enjoyment and reading self-perceived ability measures separately. We also conducted the analyses on a reading motivation composite that was computed at each wave by reverse-scoring and then averaging the two items. The results from the three analyses were highly consistent. We report the results of the analyses of the reading motivation composite in the results section and of self-perceived ability and enjoyment independently in the supplementary material.

#### Reading achievement

At age 9/10 and age 12, reading achievement was measured via the Reading Comprehension subtest of the Peabody Individual Achievement Test (PIAT; Markwardt, 1997). Children were asked to read a series of sentences and to select the one picture (out of four choices) that best depicts the meaning of the sentence. The PIAT included a total of 89 items arranged in the order of increasing difficulty. For example, one of the initial items was “Some kittens are in the bed”. The test became increasingly more complex and one of the final items was “The verdant countryside is prodigiously arable; however, a squalid domicile sullies the otherwise exquisite panorama.” Children were given up to 20 seconds to read each sentence and another 20 seconds to make their choices. A total reading achievement score was computed by summing the points across all 89 items.

### Analytic Strategies

After running descriptive and correlation analyses, we applied structural equation modeling to examine the longitudinal relations between reading achievement and reading motivation, as well as the underlying genetic and environmental etiologies of these longitudinal associations. We conducted these analyses using the OpenMx package for R (Neale et al., 2016; R Core Team, 2015).

**Figure 1 fig1:**
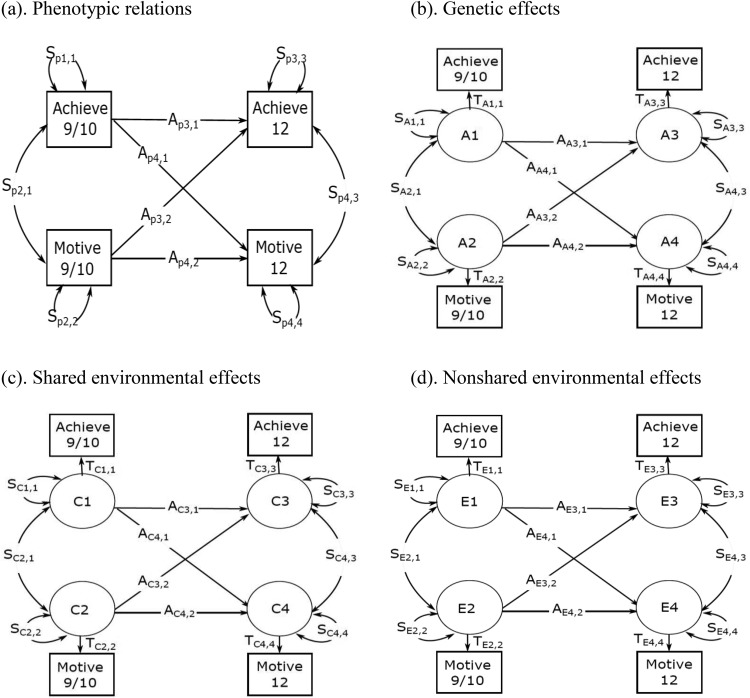
Phenotypic cross-lagged model (panel a) and ACE cross-lagged model (panel b, c, and d). S and A matrices respectively capture symmetric and asymmetric relations. T matrix captures the impact of A, C, and E components on the total phenotypic variance of each variable. In the ACE cross-lagged model, S and A matrices are further decomposed into genetic (A; panel b), shard environmental (C; panel c), and nonshared environmental (E; panel d) components. Achieve = reading achievement; motive = reading motivation; 9/10 = age 9/10; 12 = age 12.

In order to test our first hypothesis, we fitted a phenotypic cross-lagged model (Figure 1a). The cross-lagged model allows for the estimation of the strength of the link from reading motivation at age 9/10 to reading achievement at age 12, and of the opposite link from reading achievement at age 9/10 to reading motivation at age 12. The cross-lagged associations are estimated independently of the stability of the measures and their initial contemporaneous correlations. In order to formally compare the magnitude of the cross-lagged links, we constrained them to be equal. This allowed us to examine whether such constraints would worsen model fit, indicating differences in the magnitude of the paths.[Fig-anchor fig1]

We used the twin design to test our second hypothesis. The twin method allows for the examination of the relative contribution of genetic and environmental factors to the longitudinal relations between reading achievement and reading motivation. The method is based on the comparison of the concordance between monozygotic (MZ) twins, who share 100% of their genetic make-up, and dizygotic (DZ) twins, who share on average 50% of their segregating genes. Genetic and environmental influences can be calculated by comparing correlations for MZ and DZ twins for the same trait (intraclass correlations). A stronger intraclass correlation between MZ twins than between DZ twins indicates that genetic factors are involved in explaining individual differences in that trait. This allows for the decomposition of the total variance of a trait into heritability, shared environmental, and nonshared environmental influences.

Heritability (A) refers to the proportion of the phenotypic (i.e., observed) individual differences attributable to genetic influences. The remaining variance in the trait is further divided into shared and nonshared environmental influences. Shared environment (C) refers to any nongenetic influences that contribute to twin similarities. Nonshared environment (E) refers to any nongenetic influences that contribute to dissimilarities between two twins raised in the same family, and includes measurement error.

The twin method can be extended to examine the etiology of the covariance between multiple traits. Multivariate models are based on the cross-twin cross-trait correlations. Cross-twin cross-trait correlations describe the association between two traits, with twin 1’s score on the first trait correlated with twin 2’s score on the second trait. Cross-twin cross-trait correlations are computed separately for MZ and DZ twins. A higher cross-twin cross-trait correlation for MZ than for DZ twins indicates that genetic factors have a degree of influence on the phenotypic variance shared by two traits. For example, in the present study, the cross-twin cross-trait correlation between reading motivation at age 9/10 and reading achievement at age 9/10 was .22 for MZ twins and .05 for DZ twins. This suggests that genetic factors are implicated in the etiology of the covariance between reading motivation at age 9/10 and reading achievement at age 9/10.

Specifically, to test our second hypothesis we applied the ACE cross-lagged model (Figure 1b to Figure 1d). This model allowed us to examine the etiologies of the cross-lagged associations between reading motivation and reading achievement. The limitation of previously used cross-lagged models, using a multivariate Cholesky decomposition approach (see Figure 3 and Figure 4), is that the two cross-lagged paths can only be estimated in two separate models, prohibiting direct comparisons of their effects (phenotypically or etiologically). The ACE cross-lagged model used in this study overcomes this limitation by estimating all the paths within the same model.

The ACE cross-lagged model is based on the Reticular Action Model definition (McArdle & McDonald, 1984): *C = F (I—A)*^−*1*^ *S (I—A)*^*−1*′^ *F*′ where I is the identity matrix, S the matrix defining two-way relationships or symmetric relationships (i.e., variances and covariances), A is the matrix defining one-way relationships or asymmetric relationships (i.e., stability and cross-lagged paths in the case of cross-lagged model), and F is the filter matrix defining observed variables (not used here). The A and S matrices are *n* × *n* matrices, where *n* is the number of observed variables. In the ACE cross-lagged model, the twin design allows us to decompose the variance and covariance into the genetic (Figure 1b), shared environmental (Figure 1c), and nonshared environmental (Figure 1d) components, using the formulae reported below. The formulae were introduced into the model as matrix algebra to allow for the estimation of 95% confidence intervals.

**Figure 2 fig2:**
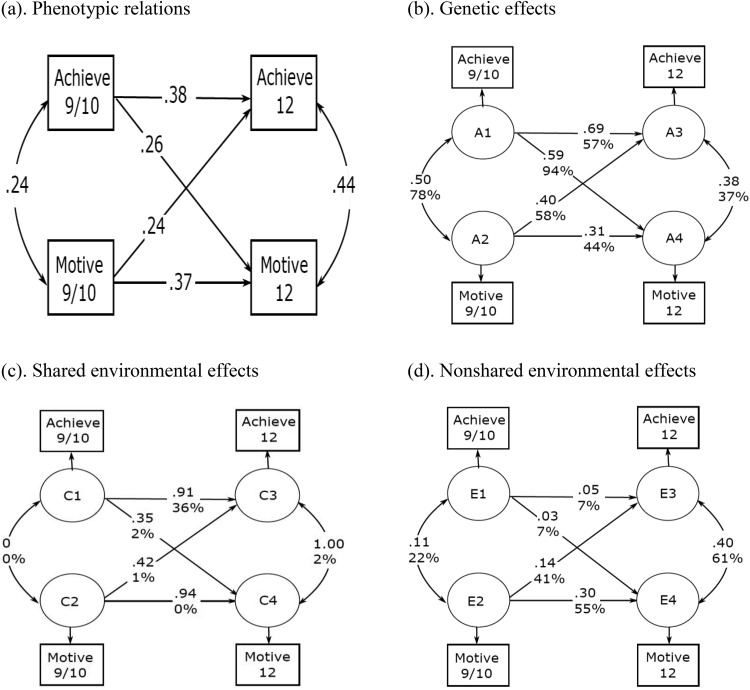
Phenotypic cross-lagged model (panel a) and ACE cross-lagged model (panel b, c, and d) with standardized path estimates. Numbers in % represent the percentage of phenotypic relations attributable to genetic, shared environmental, and nonshared environmental influences. Note that some shared environment path estimates are large whereas the corresponding % numbers are small. For example, stability for motivation in the shared environment model is .94, whereas the % number is 0. This is because shared environmental influences were very small for motivation; however, the limited shared environmental influences contributing to variance in motivation largely overlap across 2 waves, resulting in a high stability C path. However, comparing to the contribution of genes and nonshared environment, shared environmental influences were rather small, taking up around 0% of the total phenotypic stability in motivation. See Figure 1 for abbreviations.

In the ACE cross-lagged model, the twin design allows us to further decompose the variance and covariance into the genetic (Figure 2b), shared environmental (Figure 2c), and nonshared environmental (Figure 2d) components using the following formulas: *C_A_ = T_A_ (I—A_A_)^−1^ S_A_ (I—A_A_)^−1′^ T_A_′; C_C_ = T_C_ (I—A_C_)^−1^ S_C_ (I—A_C_)^−1′^ T_C_′; C_E_ = T_E_ (I—A_E_)^−1^ S_E_ (I—A_E_)^−1′^ T_E_′*, where *C_A_ + C_C_ + C_E_ = C_P_* (total observed covariance matrix). *T*_*A*_, *T*_*C*_, and *T*_*E*_ are diagonal *n* × *n* matrices, and they respectively index the impact of genetic, shared environmental, and nonshared environmental factors on the total observed variance of the variable of interest. *C*_*A*_, *C*_*C*_, and *C*_*E*_ are all constrained to 1 so that A and S matrices provide standardized relations between genetic and environmental factors. Variance components are used to define cross-twin cross-trait covariance matrices for MZ and DZ twin pairs:

$$\begin{array}{l} C_{MZ}=\left[\begin{array}{cc} C_{A}+C_{C}+C_{E} & C_{A}+C_{C}\\ C_{A}+C_{C} & C_{A}+C_{C}+C_{E} \end{array}\right]\\ C_{DZ}=\left[\begin{array}{cc} C_{A}+C_{C}+C_{E} & 0.5\times C_{A}+C_{C}\\ 0.5\times C_{A}+C_{C} & C_{A}+C_{C}+C_{E} \end{array}\right] \end{array}$$

**Figure 3 fig3:**
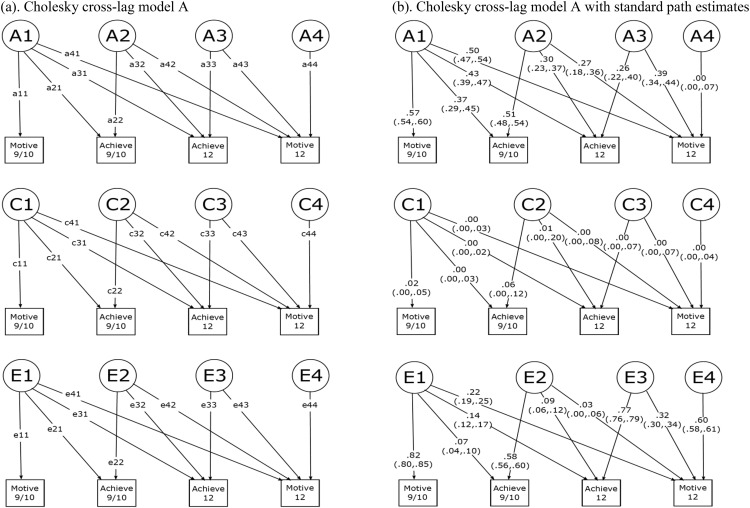
Cholesky Cross-lagged Model A. This model was used to examine the cross-lagged association between reading achievement at age 9/10 and reading motivation at age 12. See Figure 1 for abbreviations.

**Figure 4 fig4:**
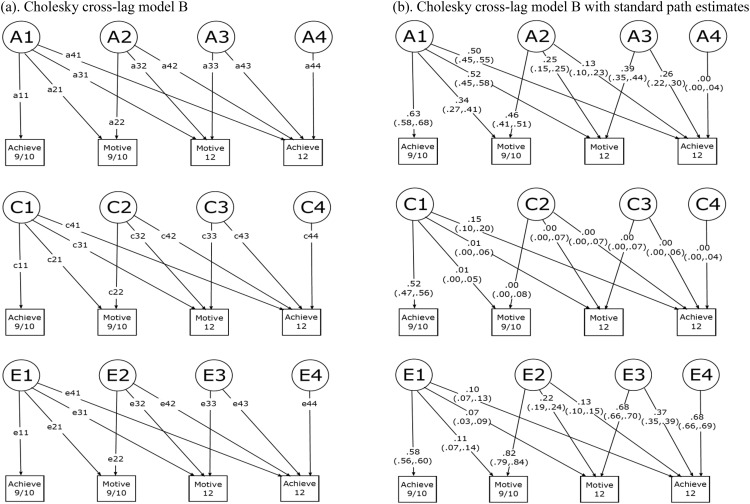
Cholesky Cross-lagged Model B. This model was used to examine the cross-lagged association between reading motivation at age 9/10 and reading achievement at age 12. See Figure 1 for abbreviations.

[Fig-anchor fig2][Fig-anchor fig3][Fig-anchor fig4]

The proportion of variance for variable *i* accounted for by the genetic, shared environmental, and nonshared environmental components is respectively estimated via dividing each variance component (*C*_*A*_, *C*_*C*_, and *C*_*E*_) by the phenotypic variance of variable i taken from the covariance matrix (*C*_*P*_) using the following formulas: *C*_*A i,i*_/*C*_*P i,i*_, *C*_*C i,i*_/*C*_*P i,i,*_ and *C*_*Ei,i*_/*C*_*P i,i.*_ The genetic, shared environment, and nonshared environment path estimates are obtained from the A and S matrices, and represent the relations between genetic and environmental factors underlying the phenotypic relations. The proportion of the observed relation between two given variables *i* and *j* that is attributable to the genetic, shared environmental, and nonshared environmental influences is estimated based on the following formula, introduced in the model as matrix algebra to allow for the estimation of 95% confidence intervals:

$$\begin{array}{l} A_{A_{i,j}}^{\%}=\frac{T_{A_{i}}A_{A_{i,j}}T_{A_{j}}}{T_{A_{i}}A_{A_{i,j}}T_{A_{j}}+T_{C_{i}}A_{C_{i,j}}T_{C_{j}}+T_{E_{i}}A_{E_{i,j}}T_{E_{j}}}\\ S_{A_{i,j}}^{\%}=\frac{T_{A_{i}}S_{A_{i,j}}T_{A_{j}}}{T_{A_{i}}S_{A_{i,j}}T_{A_{j}}+T_{C_{i}}S_{C_{i,j}}T_{C_{j}}+T_{E_{i}}S_{E_{i,j}}T_{E_{j}}}\\ A_{C_{i,j}}^{\%}=\frac{T_{C_{i}}A_{C_{i,j}}T_{C_{}}}{T_{A_{i}}A_{A_{i,j}}T_{Aj}+T_{C_{i}}A_{C_{i,j}}T_{C_{j}}+T_{E_{i}}A_{E_{i,j}}T_{E_{j}}}\\ S_{C_{i,j}}^{\%}=\frac{T_{C_{i}}S_{C_{i,j}}T_{C_{}}}{T_{A_{i}}S_{A_{i,j}}T_{Aj}+T_{C_{i}}S_{C_{i,j}}T_{C_{j}}+T_{E_{i}}S_{E_{i,j}}T_{E_{j}}}\\ A_{E_{i,j}}^{\%}=\frac{T_{E_{i}}A_{E_{i,j}}T_{E_{}}}{T_{A_{i}}A_{A_{i,j}}T_{Aj}+T_{C_{i}}A_{C_{i,j}}T_{C_{j}}+T_{E_{i}}A_{E_{i,j}}T_{E_{j}}}\\ S_{E_{i,j}}^{\%}=\frac{T_{E_{i}}S_{E_{i,j}}T_{E_{}}}{T_{A_{i}}S_{A_{i,j}}T_{Aj}+T_{C_{i}}S_{C_{i,j}}T_{C_{j}}+T_{E_{i}}S_{E_{i,j}}T_{E_{j}}} \end{array}$$

In order to validate the results obtained with the ACE cross-lagged model, we explored the same research question using the multivariate Cholesky decomposition approach, previously used to investigate the etiology of cross-lagged associations in several studies (e.g., Luo et al., 2010, 2011). When variables are entered in the appropriate order, the Cholesky model allows for the estimation of genetic and environmental influences on the variance of a single trait. In addition, it allows for the examination of the genetic and environmental factors underlying the covariance between multiple traits, including their longitudinal stabilities, contemporaneous correlations, and cross-lagged predictions. The model works similarly to a phenotypic hierarchical regression, so that the influence of one variable on another is calculated after controlling for the effect of the variables that were previously entered in the model.

As previously mentioned, the Cholesky approach only allows for the estimation of one cross-lagged path within one model. Therefore, we first examined the cross-lagged link from reading achievement at age 9/10 to reading motivation at age 12 (Cholesky cross-lag model, Figure 3a), entering the variables into the model in the following order: (1) reading motivation age 9/10, (2) reading achievement age 9/10, (3) reading achievement age 12, and (4) reading motivation age 12.

In this model, tracing paths from the factors A1, C1, and E1 it is possible to derive four sets of genetic, shared environmental, and nonshared environmental estimates for (a) the variance in reading motivation at age 9/10 (a_11_ × a_11_, c_11_ × c_11_, e_11_ × e_11_), (b) the contemporaneous covariance between reading achievement and reading motivation at 9/10 (a_11_ × a_21_, c_11_ × c_21_, e_11_ × e_21_), (c) the cross-lagged covariance between reading achievement at 9/10 and reading motivation at 12 (a_11_ × a_31_, c_11_ × c_31_, e_11_ × e_31_) (however, this cross-lagged estimate does not account for stability of reading motivation), and (d) the stability of reading achievement over time (a_11_ × a_41_, c_11_ × c_41_, e_11_ × e_41_). Tracing paths from factors A2, C2, and E2 it is possible to derive the genetic, shared environmental, and nonshared environmental estimates for (e) the residual variance of reading achievement at age 9/10 (a_22_ × a_22_, c_22_ × c_22_, e_22_ × e_22_), (f) the stability of reading achievement over time (a_22_ × a_32_, c_22_ × c_32_, e_22_ × e_32_), (g) and the cross-lagged covariance between reading achievement at age 9/10 and reading motivation at age 12 (main research interest; a_22_ × a_42_, c_22_ × c_42_, e_22_ × e_42_) independent of reading motivation at age 9/10. Tracing paths from factors A3, C3, and E3 it is possible to obtain the genetic, shared environmental, and nonshared environmental estimates for (h) the residual variance of reading achievement at age 12 (a_33_ × a_33_, c_33_ × c_33_, e_33_ × e_33_) and (i) the contemporaneous covariance between reading motivation and reading achievement at age 12 (a_33_ × a_43_, c_33_ × c_43_, e_33_ × e_43_) independent of reading motivation and reading achievement at age 9/10. Finally, A4, C4, and E4, respectively, capture the residual genetic, shared environmental, and nonshared environmental variance unique to reading motivation at age 12 after controlling for reading motivation at age 9/10 and reading achievement at both ages (a_44_ × a_44_, c_44_ × c_44_, e_44_ × e_44_).

Next, we ran a second Cholesky decomposition to examine the opposite cross-lagged link, from reading motivation at age 9/10 to reading achievement at age 12 (see Cholesky cross-lag Model B and Figure 4a). For this second model, we entered the same variables in a different order: (1) reading achievement age 9/10, (2) reading motivation age 9/10, (3) reading motivation age 12, and (4) reading achievement age 12. Similar path tracing rules were used as described above.

## Results

### Descriptive Statistics and Correlations

One twin out of each pair was randomly selected for further analyses to control for nonindependence of observation. Table 1 reports descriptive statistics. All variables were distributed widely. Distributions for reading achievement and reading motivation were similar across waves. Descriptive statistics were repeated using the other twin within the pair providing an inbuilt replication. The results were highly similar for the two samples (twin 1 and twin 2). Additionally, Table A1 reports descriptive statistics separately for MZ, same sex DZ, and opposite sex DZ twins. All zygosity groups were included in the analyses.

Phenotypic correlations between all variables are reported in Table 2. Correlations between reading motivation and reading achievement were modest at age 9/10 and age 12 (*r* = .26 and *r* = .31, respectively). The correlation between reading motivation at age 9/10 and age 12 was moderate (*r* = .50). The correlation between achievement at age 9/10 and age 12 was also moderate (*r* = .53). Prior to the genetic analyses, the effects of age and sex were controlled for, using linear regression. The same analyses were also run controlling for general intelligence, finding similar results (results of the analyses are available from the first author). All variables were Van der Waerden transformed. Van der Waerden transformation is a rank-based inverse normal transformation, which transforms the sample distribution of continuous variables to make them appear more normally distributed (see Beasley, Erickson, & Allison, 2009 for additional information). Analyses were run before and after Van der Waerden transformation. As the ranked-based transformation was found not to have an impact on the results, we ran our analyses using the transformed data.[Table-anchor tbl2]

### Twin Correlations

Table 3 presents the intraclass correlations between measures of reading achievement and reading motivation separately for MZ and DZ twins. Twin correlations were substantially larger for MZ than for DZ twins for reading motivation at both waves, indicating genetic but negligible shared environmental influences; the same was observed for reading achievement at age 12. The MZ correlation was stronger than the DZ correlation also for reading achievement at age 9/10. However, the correlation between MZ twins did not double that of DZ twins, indicating both genetic and shared environmental influences on reading achievement at age 9/10. MZ correlations for all variables were below 1, indicating nonshared environmental influences on all variables.[Table-anchor tbl3]

Table 3 also reports heritability and shared and nonshared environment estimates from univariate twin model fitting. Reading motivation at age 9/10 and age 12 was moderately heritable, with genetic factors explaining 38% and 51% of the variance, respectively. The remaining variance in reading motivation at both waves was attributable to nonshared environmental influences. Reading achievement at ages 9/10 and 12 was also moderately heritable, with genetic factors explaining 39% and 34% of the phenotypic variance, respectively. Shared environmental influences were modest for reading achievement at age 9/10 (28%), but did not contribute to individual differences in reading achievement at age 12. Nonshared environmental influences, which also include measurement error, were modest for reading achievement at age 9/10 (33%) and large for reading achievement at age 12 (66%).

Table 4 reports cross-twin cross-trait correlations for all pairwise associations. Cross-twin cross-trait correlations were generally moderate for MZ twins and weak for DZ twins, indicating genetic influence on the covariance between each pair of variables. Some of the twin correlations indicated an ADE model—decomposing the variance into additive genetic (A), nonadditive genetic (D), and nonshared environmental effects (E)—as DZ correlations were less than half the MZ correlations. However, fitting an ADE did not improve model fit indices. We therefore reported results of ACE models, as these are in line with analyses presented by previous research.[Table-anchor tbl4]

### Phenotypic Cross-Lagged Model

The phenotypic cross-lagged model allows us to explore three main concepts: correlation between variables measured at the same collection wave, stability of the variables, and cross-lagged association between different variables. Results from the phenotypic cross-lagged model are reported in Figure 2a and Table 5. The phenotypic model showed a positive modest correlation between reading motivation at age 9/10 and reading achievement at age 9/10 (*r* = .24). Reading motivation was moderately stable over time (.37), and the same was observed for reading achievement over time (.38). We observed reciprocal longitudinal links between reading motivation and reading achievement. The cross-lagged link from reading motivation at age 9/10 to reading achievement at age 12 was modest (.24). The opposite cross-lagged link from reading achievement at age 9/10 to reading motivation at age 12 was very similar (.26). Constraining the two cross-lagged paths to be equal did not result in worse model fit (χ^*2*^ = 1.76, Δ*df* = 1, *p* = .18), suggesting that the two cross-lagged paths are of similar magnitude. Finally, we observed a moderate residual positive correlation between reading motivation and reading achievement at age 12 (*r* = .44). Overall the model suggests that reading motivation at age 9/10 contributes to the variance in reading achievement at age 12 beyond the stability of achievement. Similarly and with similar strength, reading achievement at 9/10 contributed to the variance in reading motivation at age 12 beyond its stability.[Table-anchor tbl5]

### ACE Cross-Lagged Model

We tested our second hypothesis regarding the etiology of the observed longitudinal associations between reading motivation and reading achievement using the ACE cross-lagged model. The same analyses were run separately for enjoyment and self-perceived ability, and results are presented in Table A2 and A3. Results from the ACE cross-lagged model are shown in Figure 2b, 2c, 2d, and Table 5. The stability in reading motivation over time was explained by both genetic (around 44%) and nonshared environmental factors (approximately 55%). The stability in reading achievement was attributable to genetic (57%) and shared environmental influences (36%), and only a small portion of variance was explained by nonshared environmental factors (7%). The contemporaneous correlation between reading achievement and reading motivation was explained by both genetic (78%) and nonshared environmental (22%) influences. Importantly, genetic factors explained a substantial proportion of the cross-lagged link from early reading motivation to later reading achievement (58%). The remaining variance in this cross-lagged link was attributable to nonshared environment influences. The cross-lagged link from reading achievement at age 9/10 to reading motivation at age 12 was almost entirely explained by genetic factors (94%), with shared and nonshared environment explaining a negligible part of the covariance (2% and 4%, respectively). Finally, genetic factors, shared environmental factors, and nonshared environmental factors respectively accounted for 37%, 2%, and 61% of the residual contemporaneous correlation between reading motivation and reading achievement at age 12.

### Cholesky Decomposition Model

We reanalyzed the data using the traditional Cholesky decomposition approach. Standardized path estimates of Cholesky cross-lag Model A and B are shown in Figure 3b and Figure 4b. Contemporaneous correlations, stability, and cross-lagged prediction derived from the standardized path estimates are shown in Table 6. Overall, the results obtained fitting the Cholesky decomposition models were consistent with those obtained with the ACE cross-lagged model. Pertinent to our main research questions, reading achievement and reading motivation reciprocally predicted each other longitudinally after accounting for their stabilities and contemporaneous correlations. Similarly to what we observed using the ACE cross-lagged model, the link from reading motivation at age 9/10 to reading achievement at age 12 was explained by both genetic (35%) and nonshared environmental (65%) factors, and the link from reading achievement at age 9/10 to reading motivation at age 12 was almost entirely explained by genetic influences (88%), with the remaining variance explained by nonshared environmental factors (12%).[Table-anchor tbl6]

## Discussion

Using a genetically informative design, the present study tested two main hypotheses: (1) that the longitudinal relation between reading motivation and reading achievement is reciprocal, with cross-lagged links characterized by similar effect sizes; and (2) that both genetic and nonshared environmental factors contribute to the etiology of the longitudinal association between reading motivation and reading achievement.

To address the first hypothesis, we ran a phenotypic cross-lagged model. In order to test the second hypothesis we fitted a novel quantitative genetic model, the ACE cross-lagged model. Unlike other models that had been previously used in the literature (e.g., the Cholesky decomposition method), the ACE cross-lagged model allows us to examine the etiologies of all cross-lagged links within the same model, allowing to compare the effect sizes of the longitudinal links and taking into account the stability of both achievement and motivation over time. To validate the results obtained with the novel ACE cross-lagged model, we fitted a multivariate Cholesky decomposition, which has been previously used to estimate the etiology of cross-lagged links. Results were found to be consistent between the two approaches. Because we estimated the effects of all associations within one model (the ACE cross-lagged model), we were able to directly compare the effects of the two cross-lagged links (from reading motivation at age 9/10 to reading achievement at age 12, and the opposite link from reading achievement at age 9/10 to reading motivation at age 12).

At the phenotypic level, results revealed a reciprocal relation between reading motivation and achievement: Early reading achievement longitudinally predicted subsequent reading motivation over and above the effects of early reading motivation; conversely, early reading motivation also statistically predicted subsequent reading achievement controlling for the effects of early reading achievement. This indicates that, compared to their peers, children with more confidence and interests in reading are more likely to become more competent readers over time, and more skilled readers are also more likely to become more confident in their ability to read and interested in reading. The effects of the two cross-lagged links were both modest and similar in magnitude, reflecting a reciprocal association between affect and cognition in the domain of reading.

These empirical findings add to the existing literature supporting the view that a reciprocal relation exists between reading achievement and reading motivation. However, previous research mostly explored the longitudinal relationship between motivation and achievement in a domain general context (Guay et al., 2003; Marsh & Martin, 2011; Muijs, 1997), or in other specific academic domains (e.g., mathematics, Luo et al., 2011). The present study provides evidence supporting a reciprocal association between motivation and achievement also in the domain of reading. This reciprocal association was also observed when reading enjoyment and reading self-perceived ability were considered separately.

Several features of the current study may have contributed to the discrepancies between the current results and those that failed to support the view that a reciprocal association exists between motivation and achievement. First, our results revealed that the cross-lagged links were modest in magnitude. Previous investigations might have had insufficient statistical power to detect such weak reciprocal relations. Second, it is also possible that the observed reciprocal link between reading achievement and reading motivation is unique to this particular developmental stage. The developmental period from 9 to 12 years old is a period shortly after when children make the transition from “learning to read” to “reading to learn” (Chall, 1983; Harlaar, Dale, & Plomin, 2007). For younger children, development in reading skills is mainly reflected in the aspects of letter and word level processing. Improvement in these reading skills may not lead to subsequent increase in reading interests that are based primarily on comprehending reading materials for aesthetic, social, or learning reasons (Morgan et al., 2008). As children get older, the main focus of reading instruction and curricula shifts to reading comprehension. Drastic improvement in children’s comprehension skills during this stage may lead to better understanding and appreciation of reading activities, which in turn drives children to further refine their skills. As a result, mutual influences between reading motivation and reading achievement may be particularly evident at this unique developmental stage. As children get older and more fluent in reading comprehension, their growth in reading achievement levels off (Francis, Shaywitz, Stuebing, Shaywitz, & Fletcher, 1996; Wang et al., 2015), and smaller changes in reading achievement over time may be increasingly harder to predict from other noncognitive constructs, including motivation. Unfortunately, because the current data are only available over a 2-year span, we are unable to explore how the motivation–achievement relation extends to other developmental periods. Future studies over an extended time are needed to investigate the dynamic nature of the development of the motivation–achievement link in the domain of reading.

In addition to phenotypic associations, we also investigated the genetic and environmental etiologies of reading motivation and achievement and of their cross-lagged links. The etiology of individual differences in reading achievement at age 9/10 was attributable in similar parts to genetic (39%), shared (28%), and nonshared (33%) environmental influences. Variation in reading achievement at age 12 was explained moderately by genetic (34%) and mostly by nonshared environmental influences (66%). Individual differences in reading motivation at both collection waves were largely accounted for by nonshared (child specific rather than family wide) environmental factors (∼65%). The contribution of genetic factors was moderate. This is in line with a recent large international twin study that found that around 60% of individual differences in motivation in several other academic subjects could be attributed to nonshared environmental factors, and approximately 40% of the variance to genetic influences (Kovas et al., 2015).

Although nonshared environmental factors explained a substantial portion of variance in reading motivation and reading achievement at both ages, the cross-lagged links between them were largely genetic in origin. It is possible that children at genetic risk of poorer reading abilities experience more obstacles in learning to read and subsequently become more avoidant of reading activities (Harlaar, Deater-Deckard, Thompson, DeThorne, & Petrill, 2011). As a result, the less they read, the less pleasure and confidence they gain from reading. Similarly, development in reading achievement not only stemmed from genetic and environmental influences specific to reading, but was partially attributable to motivational processes by means of genetic influences.

It is important to consider that genes and environments do not operate independently. Therefore, the A, C, and E components in the variance–covariance decomposition models need to be interpreted in light of the dynamic interplay between genes and environments, which is subsumed under these variance components. Two types of gene–environment interplay may be at work: gene–environment correlation and gene by environment interaction. For example, children who have a genetic predisposition for high reading motivation may actively seek out reading activities, which in turn provide them with opportunities to practice and improve their reading skills. This process is known as active gene–environment correlation (Plomin, DeFries, & Loehlin, 1977). Alternatively, children with a genetic predisposition for good reading skills may elicit more praise and recognition from their parents and teachers, which further fosters their interests and confidence in reading activities—a process known as evocative gene–environment correlation (Plomin et al., 1977; Tucker-Drob & Harden, 2012a). Although the present results point to the possibilities of such gene–environment correlations, the current analyses do not allow us to disentangle these dynamic processes from the variance components estimation. In order to identify these gene–environment correlations, future studies should focus on examining whether relevant environmental experiences mediate the longitudinal relations between motivation and achievement through genetic pathways (Tucker-Drob, in press).

Genetically influenced individual differences drive the dynamic gene–environment correlation processes, but the existence of adequate opportunities in the environment is a necessary condition for such processes (Tucker-Drob, in press). Children who are genetically disposed to high reading motivation can only practice their reading skills when reading materials and opportunities are available to them; genetically influenced better reading skills may not result in more motivation to read without proper feedback from parents and teachers. Limitations in environment may constrain the “realization of genetic potentials,” whereas optimal environmental inputs may facilitate the translation from genetic advantage to desirable outcomes (Taylor, Roehrig, Soden Hensler, Connor, & Schatschneider, 2010; Tucker-Drob & Harden, 2012a). This process, in which environment moderates genetic effects on outcomes, is known as gene by environment interaction.

Another layer of complexity of gene–environment interplay is that environment is usually not randomly assigned to each individual (Scarr, 1996); rather, those with more genetic risks associated with poor reading abilities and low reading motivation are also potentially under more environmental risks as well (e.g., lack of supporting environment and positive feedback)–a process known as passive gene–environment correlation. These negative gene–environment feedback processes may explain why improving reading skills and reading motivation in at-risk children can be difficult (Morgan et al., 2008).

One limitation of the present study is that we focused on a specific aspect of reading achievement, reading comprehension, not considering other skills, such as reading fluency. In the same sample, reading comprehension was found to be less heritable than all other reading measures, including reading fluency (Kovas, Haworth, Dale, & Plomin, 2007). Our focus on reading comprehension may explain the discrepancy between our heritability estimates for reading achievement and those reported in the literature, which are usually higher (Kovas et al., 2007). Similarly, the present study specifically focused on the enjoyment and self-perceived ability aspect of motivation. Reading motivation is a multidimensional construct (Baker & Wigfield, 1999), and different aspects of reading motivation may be related to reading achievement via distinct mechanisms. Therefore, the present findings may not generalize to the relations between other reading cognition and other dimensions of reading motivation. For example, a recent study on a sample of 10-year old U.S. twins used a composite reading motivation score that comprises several different motivation dimensions (i.e., reading self-efficacy, reading curiosity, reading for challenges, reading for recognition, and reading for grades), and found that the concurrent association between reading comprehension and the multidimensional reading motivation was mostly accounted for by nonshared environmental influences (Schenker & Petrill, 2015).

Additionally, our reading motivation measure comprised only 2 items, which did not allow us to fully assess its psychometric properties. A short measure is likely to have lower reliability as compared to other reading motivation measures (e.g., Motivation for Reading Questionnaire; Wigfield, Guthrie, & McGough, 1996). Lower reliability may lead to underestimation of relations between constructs and overestimation of nonshared environmental influences. Therefore, replication of the present results using other measures of reading motivation is needed.

As mentioned earlier, the current data are only available at 2 time points over a 2-year span, which does not allow us to generalize the present findings to other developmental periods. Another drawback for a 2-wave cross-lagged design is that we are unable to examine the goodness of fit of our model to the data in the phenotypic cross-lagged model. Genetically sensitive studies with more repeated assessments on achievement and motivation over an extended time are needed in order to decipher the etiology of the dynamic achievement–motivation transactions.

A further limitation of the present study is that it does not allow for the identification of the potential mechanisms underlying the observed genetic associations. In fact, genetic associations could indicate that the same genes influence variation in both reading motivation and reading achievement, a concept known as pleiotropy. Alternatively, the observed genetic association might reflect genetic causality—genetic influences have an effect on one trait, for example reading motivation, and in turn reading motivation influences another trait, for example reading achievement (Ligthart & Boomsma, 2012). Our analysis does not allow disentangling between these two.

To sum up, the present study was the first in the literature to explore the longitudinal relations between achievement and motivation in the domain of reading using a genetically sensitive design. Findings from the phenotypic analyses indicated that reading motivation statistically predicted later reading achievement and reading achievement also statistically predicted subsequent reading motivation; these cross-lagged effects are similar in size, and both are independent the effects of initial reading achievement and motivation. The present findings also indicated that the longitudinal links between reading motivation and achievement primarily stem from genetic differences among individuals. The same was observed when two different aspects of the reading motivation construct, enjoyment and self-perceived ability, were considered separately. This indicates that similar mechanisms account for the longitudinal association between the two aspects of motivation and reading achievement. The specific genetic factors involved are yet to be discovered. However, acknowledging that genetic differences among people are the primary drive in this relation represents a step forward toward understanding the mechanisms underlying the association between the cognitive and affective processes implicated in reading development.

## Supplementary Material

10.1037/dev0000209.supp

## Figures and Tables

**Table 1 tbl1:** Descriptive Statistics for Reading Motivation and Achievement

Descriptives	Motivation 9/10	Motivation 12	Achievement 9/10	Achievement 12
N*	3363	5876	3095	5521
Mean	4.16	3.99	46.19	57.30
Std. Deviation	.85	.87	13.53	11.13
Skewness (std. error)	−.97 (.04)	−.74 (.03)	−.35 (.04)	−.65 (.03)
Kurtosis (std. error)	.59 (.08)	.22 (.06)	−.13 (.09)	.46 (.07)
Minimum	1.00	1.00	1.00	1.00
Maximum	5.00	5.00	80.00	81.00
*Note*. N = sample size.				
* one twin out of each pair was selected to control for non-independence of observation.				

**Table 2 tbl2:** Correlation Between Main Study Variables

Variables	1	2	3	4
1. Motivation 9/10	1	.51**	.26**	.23**
N	3363	2680	2516	2374
2. Motivation 12		1	.36**	.31**
N		5874	2433	4750
3. Achievement 9/10			1	.531**
N			3095	2272
4. Achievement 12				1
N				5521
*Note.* N = pairwise sample size. One twin was randomly selected out of each pair to control for non-independence of observation.				
** *p* < .01.				

**Table 3 tbl3:** Intraclass Correlations and Univariate Estimates for Genetic (A), Shared (C), and Nonshared (E) Environmental Influences on Reading Motivation and Reading Achievement

Variable	*r*MZ	*r*DZ	A (CIs)	C (CIs)	E (CIs)
Achievement 9/10	.67	.47	.39 (.30–.48)	.28 (.20–.35)	.33 (.30–.36)
Motivation 9/10	.42	.10	.38 (.33–.42)	—	.62 (.58–.67)
Achievement 12	.35	.15	.34 (.30–.37)	—	.66 (.63–.70)
Motivation 12	.56	.14	.51 (.48–.53)	—	.49 (.47–.52)
*Note*. Twin correlations and univariate estimates were obtained after regressing for age and sex. CIs = 95% confidence intervals.					

**Table 4 tbl4:** Cross-Twin Cross-Trait Correlations

Pairs of variables	rMZ	rDZ
Motivation 9/10 & Achievement 9/10	.22	.05^ns^
Motivation 9/10 & Achievement 12	.33	.05^ns^
Achievement 9/10 & Achievement 12	.38	.14
Achievement 9/10 & Motivation 12	.39	.15
Motivation 12 & Achievement 12	.55	.13
*Note.* Cross-twin cross-trait correlations for all pairs of variables were obtained after regressing for age and sex.		
^ns^ *p* > .05.		

**Table 5 tbl5:** Phenotypic Cross-Lagged Model and ACE Cross-Lagged Model: Model Fit Indices, Standardized Path Estimates, and Percentage of Variance Attributable to Genetic (A), Shared Environmental (C), and Nonshared Environmental (E) Influences

Path	Phenotypic	A	C	E	A(%)	C(%)	E(%)
Contemporaneous correlation	.24	.50	.00	.11	78%	0%	22%
Motivation 9/10 ⇔ Achievement 9/10	(.22, .26)	(.49, .59)	(−.02, 1.02)	(.06, .17)	(75, 89)%	(0, 16)%	(10, 34)%
Contemporaneous residual correlation	.44	.38	1.00	.40	37%	2%	61%
Motivation 12 ⇔ Achievement 12	(.43, .46)	(.25, .39)	(.84, 1.01)	(.39, .44)	(26, 37)%	(0, 6)%	(60, 70)%
Stability	.37	.31	.94	.30	44%	0%	55%
Motivation 9/10 ⇒ Motivation 12	(.35, .39)	(.18, .31)	(.00, 1.00)	(.21, .33)	(29, 57)%	(0, 0)%	(44, 72)%
Stability	.38	.69	.91	.05	57%	36%	7%
Achievement 9/10 ⇒ Achievement 12	(.36, .40)	(.44, .86)	(.91, 1.00)	(.01, .11)	(38, 57)%	(20, 52)%	(2, 15)%
Cross-lagged relation	.24	.40	.42	.14	58%	1%	41%
Motivation 9/10 ⇒ Achievement 12	(.21, .26)	(.15, .66)	(.00, .47)	(.09, .17)	(56, 75)%	(0, 9)%	(41, 49)%
Cross-lagged relation	.26	.59	.35	.03	94%	2%	7%
Achievement 9/10 ⇒ Motivation 12	(.24, .28)	(.57, .68)	(.02, .70)	(.00, .07)	(78, 98)%	(0, 2)%	(2, 15)%
Phenotypic cross-lagged model fit	−2LL(*df*) = 64171.93 (23107)	AIC = 17957.93	CFI = 1.00	RMSEA = .00			
ACE cross-lagged model fit	−2LL(*df*) = 62993.05 (23087)	AIC = 16819.05	CFI = .98	RMSEA = .01			
*Note.* All estimates were obtained after regressing for age and sex; numbers in parentheses are 95% confidence intervals; −2LL = negative 2 times log likelihood; *df* = degrees of freedom; AIC = Akaike information criterion; CFI = Bentler comparative fit index; RMSEA = root mean square error of approximation.							

**Table 6 tbl6:** Cholesky Cross-Lagged Model: Variance Components and Percentage of Phenotypic Variance Explained by Genetic (A), Shared Environment (C), and Nonshared Environment (E)

Paths	Phenotypic = A + C + E	A	C	E	A (%)	C (%)	E (%)
Contemporaneous correlation^a,b^	.26	.21	.00	.05	81%	0%	19%
Motivation 9/10 ⇔ Achievement 9/10		a_11_ × a_21_	c_11_ × c_21_	e_11_ × e_21_			
Contemporaneous residual correlation^a,b^	.35	.10	.00	.25	29%	0%	71%
Motivation 12 ⇔ Achievement 12		a_33_ × a_43_	c_33_ × c_43_	e_33_ × e_43_			
Stability^a^	.47	.29	.00	.18	62%	0%	38%
Motivation 9/10 ⇒ Motivation 12		a_11_ × a_41_	c_11_ × c_41_	e_11_ × e_41_			
Stability^b^	.46	.32	.08	.06	70%	17%	13%
Achievement 9/10 ⇒ Achievement 12		a_11_ × a_41_	c_11_ × c_41_	e_11_ × e_41_			
Cross-lagged relation^b^	.17	.06	.00	.11	35%	0%	65%
Motivation 9/10 ⇒ Achievement 12		a_22_ × a_42_	c_22_ × c_42_	e_22_ × e_42_			
Cross-lagged relation^a^	.16	.14	.00	.02	88%	0%	12%
Achievement 9/10 ⇒ Motivation 12		a_22_ × a_42_	a_22_ × a_42_	a_22_ × a_42_			
*Note.* All estimates were obtained after accounting for age and sex. Results were combined in this table in order to allow for an easier comparison with the results obtained with the ACE cross-lagged model.							
^a^ Path estimates are obtained from Cholesky cross-lagged model A in which the order of the variables are entered in the following order: motivation 9/10, achievement 9/10, achievement 12, and motivation 12. ^b^ path estimates are obtained from Cholesky cross-lagged model B in which the order of the variables are entered in the following order: achievement 9/10, motivation 9/10, motivation 12, and achievement 12.							

## References

[c1] Aunola K., Leskinen E., Onatsu-Arvilommi T., & Nurmi J. E. (2002). Three methods for studying developmental change: A case of reading skills and self-concept. British Journal of Educational Psychology, 72, 343–364. 10.1348/000709902320634447 12396310

[c2] Baker L., & Wigfield A. (1999). Dimensions of children’s motivation for reading and their relations to reading activity and reading achievement. Reading Research Quarterly, 34, 452–477. 10.1598/RRQ.34.4.4

[c3] Beasley T. M., Erickson S., & Allison D. B. (2009). Rank-based inverse normal transformations are increasingly used, but are they merited? Behavior Genetics, 39, 580–595. 10.1007/s10519-009-9281-0 19526352 PMC2921808

[c4] Bentler P. M. (1990). Comparative fit indexes in structural models. Psychological Bulletin, 107, 238–246. 10.1037/0033-2909.107.2.238 2320703

[c5] Betjemann R. S., Willcutt E. G., Olson R. K., Keenan J. M., DeFries J. C., & Wadsworth S. J. (2008). Word reading and reading comprehension: Stability, overlap and independence. Reading and Writing, 21, 539–558. 10.1007/s11145-007-9076-8

[c6] Burt S. A., McGue M., Krueger R. F., & Iacono W. G. (2005). How are parent–child conflict and childhood externalizing symptoms related over time? Results from a genetically informative cross-lagged study. Development and Psychopathology, 17, 145–165. 10.1017/S095457940505008X 15971764 PMC2245887

[c7] Calsyn R. J., & Kenny D. A. (1977). Self-concept of ability and perceived evaluation of others: Cause or effect of academic achievement? Journal of Educational Psychology, 69, 136–145. 10.1037/0022-0663.69.2.136 853147

[c8] Chall J. S. (1983). Stages of reading development. New York, NY: McGraw-Hill.

[c9] Chamorro-Premuzic T., Harlaar N., Greven C. U., & Plomin R. (2010). More than just IQ: A longitudinal examination of self-perceived abilities as predictors of academic performance in a large sample of U.K. twins. Intelligence, 38, 385–392. 10.1016/j.intell.2010.05.002 25473141 PMC4248677

[c10] Chapman J. W., & Tunmer W. E. (1997). A longitudinal study of beginning reading achievement and reading self-concept. British Journal of Educational Psychology, 67, 279–291. 10.1111/j.2044-8279.1997.tb01244.x 9376307

[c11] Davis O. S. P., Arden R., & Plomin R. (2008). G in middle childhood: Moderate genetic and shared environmental influence using diverse measures of general cognitive ability at 7, 9, and 10 years in a large population sample of twins. Intelligence, 36, 68–80. 10.1016/j.intell.2007.01.006

[c12] Deci E. L., & Ryan R. M. (2008). Self-determination theory: A macrotheory of human motivation, development, and health. Canadian Psychology/Psychologie canadienne, 49, 182–185. 10.1037/a0012801

[c13] De Naeghel J., Van Keer H., Vansteenkiste M., & Rosseel Y. (2012). The relation between elementary students’ recreational and academic reading motivation, reading frequency, engagement, and comprehension: A self-determination theory perspective. Journal of Educational Psychology, 104, 1006–1021. 10.1037/a0027800

[c14] Francis D. J., Shaywitz S. E., Stuebing K. K., Shaywitz B. A., & Fletcher J. M. (1996). Developmental lag versus deficit models of reading disability: A longitudinal, individual growth curves analysis. Journal of Educational Psychology, 88, 3–17. 10.1037/0022-0663.88.1.3

[c15] Gottschling J., Spengler M., Spinath B., & Spinath F. M. (2012). The prediction of school achievement from a behaviour genetic perspective. Results from the German twin study on cognitive ability, self-reported motivation, and school achievement (CoSMoS). Personality and Individual Differences, 53, 381–386. 10.1016/j.paid.2012.01.020

[c16] Greven C. U., Harlaar N., Kovas Y., Chamorro-Premuzic T., & Plomin R. (2009). More than just IQ: School achievement is predicted by self-perceived abilities—But for genetic rather than environmental reasons. Psychological Science, 20, 753–762. 10.1111/j.1467-9280.2009.02366.x 19470122 PMC4018661

[c17] Guay F., Marsh H. W., & Boivin M. (2003). Academic self-concept and academic achievement: Development perspectives on their causal ordering. Journal of Educational Psychology, 95, 124–136. 10.1037/0022-0663.95.1.124

[c18] Guthrie J. T., Van Meter P., McCann A., Wigfield A., Bennett L., Poundstone C. C., . . . Mitchell A. M. (1996). Growth of literacy engagement: Changes in motivations and strategies during concept-oriented reading instruction. Reading Research Quarterly, 31, 306–332. 10.1598/RRQ.31.3.5

[c19] Guthrie J. T., Wigfield A., Humenick N. M., Perencevich K. C., Taboada A., & Barbosa P. (2006). Influences of stimulating tasks on reading motivation and comprehension. The Journal of Educational Research, 99, 232–246. 10.3200/JOER.99.4.232-246

[c20] Hanscombe K. B., Trzaskowski M., Haworth C. M. A., Davis O. S. P., Dale P. S., & Plomin R. (2012). Socioeconomic status (SES) and children’s intelligence (IQ): In a UK-representative sample SES moderates the environmental, not genetic, effect on IQ. PLoS ONE, 7, e30320 10.1371/journal.pone.0030320 22312423 PMC3270016

[c21] Harlaar N., Cutting L., Deater-Deckard K., Dethorne L. S., Justice L. M., Schatschneider C., . . . Petrill S. A. (2010). Predicting individual differences in reading comprehension: A twin study. Annals of Dyslexia, 60, 265–288. 10.1007/s11881-010-0044-7 20814768 PMC2981603

[c22] Harlaar N., Dale P. S., & Plomin R. (2007). From learning to read to reading to learn: Substantial and stable genetic influence. Child Development, 78, 116–131. 10.1111/j.1467-8624.2007.00988.x 17328696

[c23] Harlaar N., Deater-Deckard K., Thompson L. A., Dethorne L. S., & Petrill S. A. (2011). Associations between reading achievement and independent reading in early elementary school: A genetically informative cross-lagged study. Child Development, 82, 2123–2137. 10.1111/j.1467-8624.2011.01658.x 22026450 PMC3610326

[c24] Hart S. A., Petrill S. A., & Thompson L. A. (2010). A factorial analysis of timed and untimed measures of mathematics and reading abilities in school aged twins. Learning and Individual Differences, 20, 63–69. 10.1016/j.lindif.2009.10.004 20161680 PMC2821061

[c25] Haworth C. M., Davis O. S., & Plomin R. (2013). Twins Early Development Study (TEDS): A genetically sensitive investigation of cognitive and behavioral development from childhood to young adulthood. Twin Research and Human Genetics, 16, 117–125. 10.1017/thg.2012.91 23110994 PMC3817931

[c26] Kaplan E., Fein D., Kramer J., Delis D., & Morris R. (1999). WISC-III-PI manual. San Antonio, TX: The Psychological Corporation.

[c27] Kovas Y., Garon-Carrier G., Boivin M., Petrill S. A., Plomin R., Malykh S. B., . . . Vitaro F. (2015). Why children differ in motivation to learn: Insights from over 13,000 twins from 6 countries. Personality and Individual Differences, 80, 51–63. 10.1016/j.paid.2015.02.006 26052174 PMC4372262

[c28] Kovas Y., Haworth C. M. A., Dale P. S., & Plomin R. (2007). The genetic and environmental origins of learning abilities and disabilities in the early school years. Monographs of the Society for Research in Child Development, 72, 1–144. 10.1111/j.1540-5834.2007.00453.x 17995572 PMC2784897

[c29] Kovas Y., Voronin I., Kaydalov A., Malykh S. B., Dale P. S., & Plomin R. (2013). Literacy and numeracy are more heritable than intelligence in primary school. Psychological Science, 24, 2048–2056. 10.1177/0956797613486982 24002885 PMC3834736

[c30] Ligthart L. & Boomsma D. (2012). Causes of Comorbidity: Pleiotropy or causality? Shared genetic and environmental influences on migraine and neuroticism. Twin Research and Human Genetics, 15, 158–165. 10.1375/twin.15.2.158 22856357

[c31] Linnenbrink E. A., & Pintrich P. R. (2003). The role of self-efficacy beliefs in student engagement and learning in the classroom. Reading & Writing Quarterly: Overcoming Learning Difficulties, 19, 119–137. 10.1080/10573560308223

[c32] Logan J. A., Hart S. A., Cutting L., Deater-Deckard K., Schatschneider C., & Petrill S. (2013). Reading development in young children: Genetic and environmental influences. Child Development, 84, 2131–2144. 10.1111/cdev.12104 23574275 PMC3773299

[c33] Luo Y. L., Haworth C. M., & Plomin R. (2010). A novel approach to genetic and environmental analysis of cross-lagged associations over time: The cross-lagged relationship between self-perceived abilities and school achievement is mediated by genes as well as the environment. Twin Research and Human Genetics, 13, 426–436. 10.1375/twin.13.5.426 20874463 PMC3819564

[c34] Luo Y. L., Kovas Y., Haworth C. M., & Plomin R. (2011). The etiology of mathematical self-evaluation and mathematics achievement: Understanding the relationship using a cross-lagged twin study from age 9 to 12. Learning and Individual Differences, 21, 710–718. 10.1016/j.lindif.2011.09.001 22102781 PMC3217262

[c35] Markwardt F. C. (1997). Peabody Individual Achievement Test-Revised/Normative Update. Minneapolis, MN: Pearson Assessments.

[c36] Marsh H. W., & Martin A. J. (2011). Academic self-concept and academic achievement: Relations and causal ordering. British Journal of Educational Psychology, 81, 59–77. 10.1348/000709910X503501 21391964

[c37] Marsh H. W., Trautwein U., Ludtke O., Koller O., & Baumert J. (2006). Integration of multidimensional self-concept and core personality constructs: Construct validation and relations to well-being and achievement. Journal of Personality, 74, 403–456. 10.1111/j.1467-6494.2005.00380.x 16529582

[c38] McArdle J. J., & McDonald R. P. (1984). Some algebraic properties of the Reticular Action Model for moment structures. British Journal of Mathematical & Statistical Psychology, 37, 234–251. 10.1111/j.2044-8317.1984.tb00802.x 6509005

[c39] Morgan P. L., & Fuchs D. (2007). Is there a bidirectional relationship between children’s reading skills and reading motivation? Exceptional Children, 73, 165–183. 10.1177/001440290707300203

[c40] Morgan P. L., Fuchs D., Compton D. L., Cordray D. S., & Fuchs L. S. (2008). Does early reading failure decrease children’s reading motivation? Journal of Learning Disabilities, 41, 387–404. 10.1177/0022219408321112 18768772

[c41] Muijs R. D. (1997). Predictors of academic achievement and academic self-concept: A longitudinal perspective. British Journal of Educational Psychology, 67, 263–277. 10.1111/j.2044-8279.1997.tb01243.x 9376306

[c42] National Assessment of Educational Progress (2003). Mathematics: Student Background Questionnaire. Retrieved October 1, 2003 from http://nces.ed.gov/nationsreportcard/pdf/bgq/student/math/Z1mb1.pdf

[c43] Neale M. C., Hunter M. D., Pritikin J. N., Zahery M., Brick T. R., Kirkpatrick R. M., . . . Boker S. M. (2016). OpenMx 2.0: Extended structural equation and statistical modeling. Psychometrika, 81, 535–549. 25622929 10.1007/s11336-014-9435-8 PMC4516707

[c44] Nurmi J. E., & Aunola K. (2005). Task-motivation during the first school years: A person-oriented approach to longitudinal data. Learning and Instruction, 15, 103–122. 10.1016/j.learninstruc.2005.04.009

[c101] Oakhill J. V., & Cain K. (2012). The precursors of reading ability in young readers: Evidence from a four-year longitudinal study. Scientific Studies of Reading, 16, 91–121.

[c45] Oliver B. R., & Plomin R. (2007). Twins’ Early Development Study (TEDS): A multivariate, longitudinal genetic investigation of language, cognition and behavior problems from childhood through adolescence. Twin Research and Human Genetics, 10, 96–105. 10.1375/twin.10.1.96 17539369

[c46] Plomin R. (2014). Genotype–environment correlation in the era of DNA. Behavior Genetics, 44, 629–638. 10.1007/s10519-014-9673-7 25195166 PMC4234822

[c47] Plomin R., DeFries J. C., & Loehlin J. C. (1977). Genotype–environment interaction and correlation in the analysis of human behavior. Psychological Bulletin, 84, 309–322. 10.1037/0033-2909.84.2.309 557211

[c48] Plomin R., DeFries J. C., McClearn G. E., & McGuffin P. (2008). Behavioral genetics (5th ed.). New York, NY: Worth.

[c49] Raven J. C., Court J. H., & Raven J. (1996). Manual for Raven’s Progressive Matrices and Vocabulary Scales. New York, NY: Oxford University Press.

[c50] R Core Team (2015). R: A language and environment for statistical computing. Vienna, Austria: R Foundation for Statistical Computation Retrieved from http://www.r-project.org

[c51] Ritchie S. J., & Bates T. C. (2013). Enduring links from childhood mathematics and reading achievement to adult socioeconomic status. Psychological Science, 24, 1301–1308. 10.1177/0956797612466268 23640065

[c52] Ryan R. M., & Deci E. L. (2000). Self-determination theory and the facilitation of intrinsic motivation, social development, and well-being. American Psychologist, 55, 68–78. 10.1037/0003-066X.55.1.68 11392867

[c53] Scarr S. (1996). How people make their own environments: Implications for parents and policy makers. Psychology, Public Policy, and Law, 2, 204–228. 10.1037/1076-8971.2.2.204

[c54] Schenker V. J., & Petrill S. A. (2015). Overlapping genetic and child-specific nonshared environmental influences on listening comprehension, reading motivation, and reading comprehension. Journal of Communication Disorders, 57, 94–105. 10.1016/j.jcomdis.2015.07.006 26321677 PMC4609295

[c55] Schutte N., & Malouff J. M. (2004). University student reading preferences in relation to the big five personality dimensions. Reading Psychology, 25, 273–295. 10.1080/02702710490522630

[c56] Skaalvik E. M., & Valas H. (1999). Relations among achievement, self-concept, and motivation in mathematics and language arts: A longitudinal study. Journal of Experimental Education, 67, 135–149. 10.1080/00220979909598349

[c57] Smith P., Fenandes C., & Strand S. (2001). Cognitive abilities test 3: Technical manual. Windsor, U.K.: NFERNelson.

[c58] Spinath B., Spinath F. M., Harlaar N., & Plomin R. (2006). Predicting school achievement from general cognitive ability, self-perceived ability, and intrinsic value. Intelligence, 34, 363–374. 10.1016/j.intell.2005.11.004

[c59] Steiger J. H. (1990). Structural model evaluation and modification: An interval estimation approach. Multivariate Behavioral Research, 25, 173–180. 10.1207/s15327906mbr2502_4 26794479

[c60] Steinmayr R., & Spinath B. (2009). The importance of motivation as a predictor of school achievement. Learning and Individual Differences, 19, 80–90. 10.1016/j.lindif.2008.05.004

[c61] Stipek D. J. (1996). Motivation and instruction In Berliner D. C. & Calfee R. C. (Eds.), Handbook of educational psychology (pp. 85–113). New York, NY: Macmillan.

[c62] Taylor J., Roehrig A. D., Soden Hensler B., Connor C. M., & Schatschneider C. (2010). Teacher quality moderates the genetic effects on early reading. Science, 328, 512–514. 10.1126/science.1186149 20413504 PMC2905841

[c63] Tucker-Drob E. M. (in press). Motivational factors as mechanisms of gene–environment transactions in cognitive development and academic achievement In Elliot A., Dweck C., & Yeager D. (Eds.), Handbook of competence and motivation, second edition: Theory and application. New York, NY: Guilford Press.

[c64] Tucker-Drob E. M., & Harden K. P. (2012a). Early childhood cognitive development and parental cognitive stimulation: Evidence for reciprocal gene–environment transactions. Developmental Science, 15, 250–259. 10.1111/j.1467-7687.2011.01121.x 22356180 PMC3296290

[c65] Tucker-Drob E. M., & Harden K. P. (2012b). Intellectual interest mediates gene × socioeconomic status interaction on adolescent academic achievement. Child Development, 83, 743–757. 22288554 10.1111/j.1467-8624.2011.01721.x PMC3305825

[c66] Wang Z., Soden B., Deater-Deckard K., Lukowski S., Schenker V., Willcutt E., . . . Petrill S. A. (2015). Development in reading and math in children from different SES backgrounds: The moderating role of child temperament. Developmental Science. Advance online publication 10.1111/desc.12380 PMC4916056 26689998

[c102] Warmington M., & Hulme C. (2012). Phoneme awareness, visual-verbal paired-associate learning, and rapid automatized naming as predictors of individual differences in reading ability. Scientific Studies of Reading, 16, 45–62.

[c67] Wechsler D. (1992). Wechsler Intelligence Scale for Children—Third Edition UK (WISC-IIIUK) Manual. London, England: The Psychological Corporation.

[c68] Wigfield A. (1997). Reading motivation: A domain-specific approach to motivation. Educational Psychologist, 32, 59–68. 10.1207/s15326985ep3202_1

[c69] Wigfield A., & Eccles J. S. (2000). Expectancy-value theory of achievement motivation. Contemporary Educational Psychology, 25, 68–81. 10.1006/ceps.1999.1015 10620382

[c70] Wigfield A., Eccles J. S., Mac Iver D. M., Reuman D. A., & Midgley C. (1991). Transitions during early adolescence: Changes in children’s domain-specific self-perceptions and general self-esteem across the transition to junior high school. Developmental Psychology, 27, 552–565. 10.1037/0012-1649.27.4.552

[c71] Wigfield A., & Guthrie J. T. (1997). Relations of children’s motivation for reading to the amount and breadth or their reading. Journal of Educational Psychology, 89, 420–432. 10.1037/0022-0663.89.3.420

[c103] Wigfield A., Guthrie T. J., & McGough K. (1996). A questionnaire measure of reading motivations (Instructional Resource No 22). Athens, GA: National Reading Research Center.

[c72] Wigfield A., Guthrie J. T., Tonks S., & Perencevich K. C. (2004). Children’s motivation for reading: Domain specificity and instructional influences. The Journal of Educational Research, 97, 299–310. 10.3200/JOER.97.6.299-310

