# Supplementary material for: Reading Self-Perceived Ability, Enjoyment and Achievement: A Genetically Informative Study of Their Reciprocal Links Over Time
Source: Dev Psychol. 2017 Apr;53(4):698–712. doi: 10.1037/dev0000209 (PMC5363396; doi:10.1037/dev0000209)
Supplement: Supplementary file 1 [file z2p999173829so1.docx]

**Supplemental Materials**

**Reading Self-Perceived Ability, Enjoyment and Achievement: A Genetically Informative Study of Their Reciprocal Links Over Time**

**by M. Malanchini et al., 2017, *Developmental Psychology***

**http://dx.doi.org/10.1037/dev0000209**

**Appendix**

Table A1. Descriptive statistics separately for MZ, same sex (SS) DZ and opposite sex (OS) DZ twins

|  | N* | Mean | St Deviation | Skewness (St error) | Kurtosis (St error) | Minimum | Maximum |
| --- | --- | --- | --- | --- | --- | --- | --- |
| Motivation 9/10 **MZ** | 1,230 | 4.16 | .84 | -1.03 (.07) | .84 (.14) | 1.00 | 5.00 |
| Motivation 9/10 **DZ SS** | 1,084 | 4.16 | .84 | -.95 (.07) | .38 (.15) | 1.00 | 5.00 |
| Motivation 9/10 **DZ OS** | 1,033 | 4.16 | .86 | -.94 (.07) | .54 (.15) | 1.00 | 5.00 |
| Reading 10 **MZ** | 1,112 | 45.47 | 13.65 | -.30 (.07) | -.06 (.14) | 1.00 | 79.00 |
| Reading 10 **DZ SS** | 985 | 46.57 | 13.56 | -.36 (.07) | -.25(.15) | 1.00 | 77.00 |
| Reading 10 **DZ OS** | 983 | 46.74 | 13.31 | -.39 (.07) | -.06 (.15) | 3.00 | 80.00 |
| Motivation 12 **MZ** | 2,107 | 3.97 | .88 | -.69 (.05) | -.05 (.10) | 1.00 | 5.00 |
| Motivation 12 **DZ SS** | 1,904 | 4.00 | .84 | -.62 (.05) | .00 (.11) | 1.00 | 5.00 |
| Motivation 12 **DZ OS** | 1,839 | 4.02 | .87 | -.65 (.05) | -.26 (.11) | 1.00 | 5.00 |
| Reading 12 **MZ** | 1,990 | 56.62 | 11.13 | -.52 (.05) | -.05(.11) | 3.00 | 79.00 |
| Reading 12 **DZ SS** | 1,764 | 57.78 | 11.10 | -.73 (.06) | .88 (.11) | 3.00 | 81.00 |
| Reading 12 **DZ OS** | 1,739 | 57.67 | 11.01 | -.71 (.05) | .72 (.11) | 1.00 | 79.00 |

* = 1 twin out of each pair was randomly selected.

Table A2. Phenotypic and ACE cross-lagged model for the longitudinal association between reading self-perceived ability (SPA) and reading achievement: Model fit indices, standardized path estimates, and percentage of variance attributable to genetic (A), shared environmental (C), and nonshared environmental (E) influences.

| Path | Phenotypic | A | C | | E | A(%) | | C(%) | | E(%) |
| --- | --- | --- | --- | --- | --- | --- | --- | --- | --- | --- |
| Contemporaneous correlation  SPA 9/10⬄Achievement 9/10 | 0.24  (0.23, 0.24) | 0.53  (0.52, 0.53) | 0.02  (0.02, 0.36) | | 0.10  (0.04, 0.18) | 79%  (70, 94)% | | 1%  (0%, 17%) | | 20%  (6, 32)% |
| Contemporaneous residual correlation  SPA 12⬄Achievement 12 | 0.37  (0.36, 0.38) | 0.37  (0.37, 0.63) | -0.01  (-0.01, 0.02) | | 0.31  (0.30, 0.34) | 42%  (36, 48)% | | 0%  (0, 0)% | | 58%  (51, 76)% |
| Stability  SPA 9/10⇨SPA 12 | 0.24  (0.24, 0.26) | 0.08  (0.07, 0.08) | 0.02  (0.02, 1.00) | | 0.24  (0.18, 0.24) | 19%  (0, 33)% | | 0%  (0, 0)% | | 81%  (80, 84)% |
| Stability  Achievement 9/10⇨Achievement 12 | 0.39  (0.37, 0.41) | 0.71  (0.52, 0.73) | 0.93  (0.76, 0.95) | | 0.06  (0.06, 0.07) | 59%  (58, 79)% | | 33%  (21, 44)% | | 8%  (2, 9)% |
| Cross-lagged relation  SPA 9/10⇨Achievement 12 | 0.17  (0.15, 0.19) | 0.29  (0.23, 0.75) | 0.00  (0.00, 0.07) | | 0.10  (0.10, 0.10) | 58%  (57, 58)% | | 0%  (0, 1)% | | 42%  (17, 82)% |
| Cross-lagged relation  Achievement 9/10⇨SPA 12 | 0.28  (0.27, 0.30) | 0.67  (0.50, 0.79) | 0.14  (0.02, 1.00) | | 0.05  (0.04, 0.10) | 93%  (93, 100)% | | 0%  (0, 7)% | | 7%  (2, 7)% |
| Phenotypic cross-lagged model fit | -2LL(df) = 90361.5 (33705) | | | AIC = 22951.55 | | | CFI = 1.00 | | RMSEA = 0.00 | |
| ACE cross-lagged model fit | -2LL(df) = 88287.8 (33685) | | | AIC =20917.87 | | | CFI =0.98 | | RMSEA = 0.01 | |

Note. All estimates were obtained after regressing for age and sex; number in parentheses are 95% confidence interval; -2LL = negative 2 times log likelihood; df = degrees of freedom; AIC = Akaike information criterion; CFI = Bentler comparative fit index; RMSEA = root mean square error of approximation.

Table A3. Phenotypic and ACE cross-lagged model for the longitudinal association between reading enjoyment and reading achievement: Model fit indices, standardized path estimates, and percentage of variance attributable to genetic (A), shared environmental (C), and nonshared environmental (E) influences.

| Path | Phenotypic | A | C | | E | A(%) | | C(%) | | E(%) |
| --- | --- | --- | --- | --- | --- | --- | --- | --- | --- | --- |
| Contemporaneous correlation  Enjoyment 9/10⬄Achievement 9/10 | 0.17  (0.16, 0.17) | 0.31  (0.28, 0.32) | 0.00  (0.00, 0.03) | | 0.11  (0.07, 0.17) | 71%  (59, 79)% | | 0%  (0%, 0%) | | 29%  (29, 30)% |
| Contemporaneous residual correlation  Enjoyment 12⬄Achievement 12 | 0.40  (0.38, 0.42) | 0.31  (0.16, 0.46) | 0.00  (0.00, 1.00) | | 0.35  (0.32, 0.36) | 36%  (35, 37)% | | 0%  (0, 6)% | | 64%  (55, 65)% |
| Stability  Enjoyment 9/10⇨Enjoyment 12 | 0.37  (0.34, 0.39) | 0.48  (0.40, 0.48) | 0.00  (0.00, 1.00) | | 0.22  (0.17, 0.27) | 64%  (62, 72)% | | 0%  (0, 0)% | | 36%  (27, 44)% |
| Stability  Achievement 9/10⇨Achievement 12 | 0.40  (0.40, 0.43) | 0.82  (0.62, 0.91) | 0.91  (0.74, 1.00) | | 0.05  (0.01, 0.11) | 65%  (54, 79)% | | 29%  (17, 32)% | | 6%  (0, 6)% |
| Cross-lagged relation  Enjoyment 9/10⇨Achievement 12 | 0.22  (0.22, 0.24) | 0.33  (0.19, 0.42) | 0.00  (0.00, 1.00) | | 0.14  (0.09, 0.14) | 56%  (38, 63)% | | 0%  (0, 0)% | | 44%  (27, 65)% |
| Cross-lagged relation  Achievement 9/10⇨Enjoyment 12 | 0.23  (0.21, 0.23) | 0.49  (0.35, 0.61) | 0.00  (0.00, 1.00) | | 0.02  (0.02, 0.08) | 96%  (73, 100)% | | 0%  (0, 13)% | | 4%  (4, 14)% |
| Phenotypic cross-lagged model fit | -2LL(df) = 89153.1 (33675) | | | AIC = 21807.11 | | | CFI = 1.00 | | RMSEA = 0.00 | |
| ACE cross-lagged model fit | -2LL(df) = 87083.9 (33653) | | | AIC =19777.96 | | | CFI =0.98 | | RMSEA = 0.01 | |

Note. All estimates were obtained after regressing for age and sex; number in parentheses are 95% confidence interval; -2LL = negative 2 times log likelihood; df = degrees of freedom; AIC = Akaike information criterion; CFI = Bentler comparative fit index; RMSEA = root mean square error of approximation.
